# A comparative study of microsurgery and gamma knife radiosurgery in vestibular schwannoma evaluating tumor control and functional outcome

**DOI:** 10.1093/noajnl/vdad146

**Published:** 2023-11-11

**Authors:** Marcos Tatagiba, Sophie S Wang, Ahmed Rizk, Florian Ebner, Albertus T C J van Eck, Georgios Naros, Gerhard Horstmann

**Affiliations:** Department of Neurosurgery, Eberhard Karls University, Tubingen, Germany; Department of Neurosurgery, Eberhard Karls University, Tubingen, Germany; Department of Neurosurgery, Eberhard Karls University, Tubingen, Germany; Department of Neurosurgery, Eberhard Karls University, Tubingen, Germany; Gamma Knife Center, Krefeld, Germany; Department of Neurosurgery, Eberhard Karls University, Tubingen, Germany; Gamma Knife Center, Krefeld, Germany

**Keywords:** acoustic neuroma, microsurgery, outcome, stereotactic radiosurgery, tumor recurrence, vestibular schwannoma

## Abstract

**Background:**

Both stereotactic radiosurgery (*SRS*) and microsurgical resection (*SURGERY*) are available as treatment options for sporadic vestibular schwannoma (VS). There are very few direct comparative studies comparing both treatment modalities in large cohorts allowing detailed subgroup analysis. This present study aimed to compare the nuances in the treatment of VS by *SURGERY* and *SRS* in 2 highly specialized neurosurgical centers.

**Methods:**

This is a retrospective bicentric cohort study. Data from patients treated between 2005 and 2011 were collected retrospectively. Recurrence-free survival (RFS) was assessed radiographically by contrast-enhanced magnetic resonance imaging.

**Results:**

The study population included *N* = 901 patients with a mean follow-up of 7 years. Overall, the incidence of recurrence was 7% after *SURGERY*, and 11% after *SRS* with superior tumor control in *SURGERY* in the Kaplan–Meier-analysis (*P* = 0.031). In small tumors (Koos I and II), tumor control was equivalent in both treatment arms. In large VS (Koos III and IV), however, RFS was superior in *SURGERY*. The extent of resection correlated with RFS (*P* < .001). Facial and hearing deterioration was similar in both treatment arms in small VS, but more pronounced in *SURGERY* of large VS. Tinnitus, vertigo, imbalance, and trigeminal symptoms were more often improved by *SURGERY* than *SRS*.

**Conclusions:**

*SRS* can achieve similar tumor control compared to *SURGERY* in smaller VS (Koos I and II)—with similar postinterventional morbidities. In large VS (Koos III and IV), long-term tumor control of *SRS* is inferior to *SURGERY*. Based on these results, we suggest that if combination therapy is chosen, the residual tumor should not exceed the size of Koos II.

**Key Points**

- Tumor control is comparable in small VS (Koos I and II), but *SURGERY* is superior for large VS (Koos III and IV).
- Tinnitus, vertigo, imbalance, and trigeminal symptoms are more likely to improve after *SURGERY*.
- If combination therapy is chosen, the postsurgical residual tumor should not exceed the size of Koos II.

**Importance of the Study**

The level of evidence to provide treatment recommendations for vestibular schwannoma (VS) is remarkably low. Both stereotactic radiosurgery (*SRS*) and microsurgical resection (*SURGERY*) are available as treatment options for VS. However, there are very few direct comparative studies comparing both treatment modalities in large cohorts allowing detailed subgroup analysis. More importantly, as the choice of treatment modality for VS has been regarded to be controversial, we have identified clear parameters for VS patients to benefit from *SURGERY,* like tumor size (Koos III and IV), tinnitus, trigeminal, and vertigo symptoms. In large tumors (Koos III and IV), *SRS* is not able to assure the same tumor control as *SURGERY*. This study adds valuable information to the current discussion on combination therapies: According to our data, residual tumor volume (TV) should not exceed Koos II after tumor decompression, if combination therapy should achieve similar tumor control as in *SURGERY* in large VS.

Vestibular schwannomas (VS) are benign nerve sheath tumors of the vestibular portion of the eight cranial nerves.^1–3^ Its incidence has been described to be approximately 1/100 000 per year.^4^ Serial observation, stereotactic radiosurgery (*SRS*) and microsurgical resection (*SURGERY*) are all available as contemporary treatment options for sporadic VS.^5^

Since the early days of neurosurgery, surgical resection has had a leading role in VS management. In the last decades, *SRS* has emerged as a viable and accepted alternative to *SURGERY*, also offered to young and healthy patients. However, the level of evidence to provide treatment recommendations for vestibular schwannoma (VS) is remarkably low and there are very few direct comparative studies regarding treatment modalities in large cohorts that allow detailed subgroup analysis.^6^ Decompressive surgery (DS) followed by adjuvant radiotherapy has also been discussed.^7,8^ However, the evidence for the rationale for choosing and recommending either *SRS* or *SURGERY* as a monotherapy is still lacking.^6^ Better clinical factors to stratify which treatment suits the patient best, and surrogate markers for therapy success need to be identified to assign VS patients to their best possible treatment.

The impact of VS as a disease on an individual patient is multifactorial, mainly constituting of quality of life, functional outcome, and tumor control (recurrence-free survival [RFS]). Studies focusing on questionnaires have shown that the modality of treatment (*SRS*, *SURGERY*, or observation) does not result in relevant differences in patients’ quality of life, even though it has been shown that *SURGERY* yielded worse hearing and facial nerve outcomes.^5,9^ Therefore, as primary VS outcomes like hearing and facial function have been investigated thoroughly, so-called secondary symptoms like tinnitus, trigeminal symptoms, vertigo, and imbalance ought to be assessed as well.

The present study aimed to compare the nuances of the treatment of primary VS with *SURGERY* and *SRS* in 2 specialized high-volume neurosurgical centers with more than 100 treated cases per year each. It investigated tumor control in RFS, primary (hearing and facial outcome) and secondary VS symptoms (tinnitus, vertigo/imbalance, and trigeminal function) as outcome parameters.

## Methods

### Study Design and Patient Cohort

This is a retrospective bicentric cohort study. Patients were identified by a prospectively kept registry by both senior authors (MT and GH) from 2 tertiary and specialized centers involved in the treatment of vestibular schwannomas. Clinical data were then retrospectively collected for patients with primary VS treated between 2005 and 2011 to enable long-term follow-up (FU).

### Data Collection

Tumor size was classified by Koos Classification.^10^ Previously treated VS and VS associated with neurofibromatosis were systematically excluded. The clinical state of primary VS symptoms was reported by House and Brackmann (H&B)^11^ and Gardner–Robertson (G&R) scale (with H&B 1–2 and G&R 1–2 considered as good outcome).^12^ Peri-interventional complications were classified by Clavien–Dindo Classification (CDC).^13^ RFS was assessed radiographically by contrast-enhanced magnetic resonance (MR) imaging.^14,15^ The criteria for tumor recurrence/progression was progressive tumor growth in Gadolinium contrast-enhanced MR imaging (radiographic tumor control = RTC). To exclude the known phenomenon of pseudoprogression after *SRS*, patients with tumor volume (TV) increase 6 months after SRS with stable TV afterwards or TV decrease was not graded as VS recurrence/progression.^16^ The TV was measured using slice-by-slice manual contouring and supervised by multiple board-certified Gamma-Knife-Radiosurgery (GKR)-experts.

In the case of *SURGERY*, the extent of resection (EOR) was classified by first postoperative MRI (3 months postoperative): The residual contrast-enhancing tumor was defined as subtotal resection (STR), whereas gross total resection (GTR) was defined as lack of contrast-enhancement in Gadolinium-enhanced MR imaging. Secondary VS symptoms like trigeminal affection, tinnitus, and vertigo were also collected using subgroups as the following: grade 1 (intermittent symptomatic), grade 2 (persistent symptomatic), and grade 3 (invalidating symptomatic). A reduction of 1 grade was interpreted as symptom improvement and an increase in 1 grade was interpreted as symptom worsening.

The local ethics committee approved this analysis and was in accordance with the ethical standards laid down in the Declaration of Helsinki for research involving human subjects.

### Treatment Modalities

Patients treated by *SURGERY* were all operated via retrosigmoid approach using intraoperative electrophysiological monitoring by the senior neurosurgeon (MT). Patients were either operated in a semisitting or supine position, depending on tumor size (supine position for Koos I and II, and semisitting position for Koos III and IV).^17^ All VS patients in the *SRS* cohort received GKR (Elekta AB, Stockholm, Sweden) with a prescription dose of 12–13 Gy to the 65% isodose line.

### Statistical Analysis

Statistical analysis was performed in R Studio (Version 1.2) using descriptive statistics. To compare nonnumeric parameters of both groups, the chi-square test was applied. For small number sizes, Fisher-Exact *t*-Test was applied. For numeric parameters, Welch’s 2-sample *t*-test was used. Recurrence-free survival was estimated using the Kaplan–Meier method and compared between cases and controls using a log-rank test. The length of FU for recurrence-free survival was calculated from the date of surgical intervention to the date of either recurrence or the last clinical visit. Significance was defined as the probability of a 2-sided type 1 error being <5% (*P* <.05). Data are presented as mean ± SD if not indicated otherwise.

## Results

### Study Cohort

**Figure 1.**
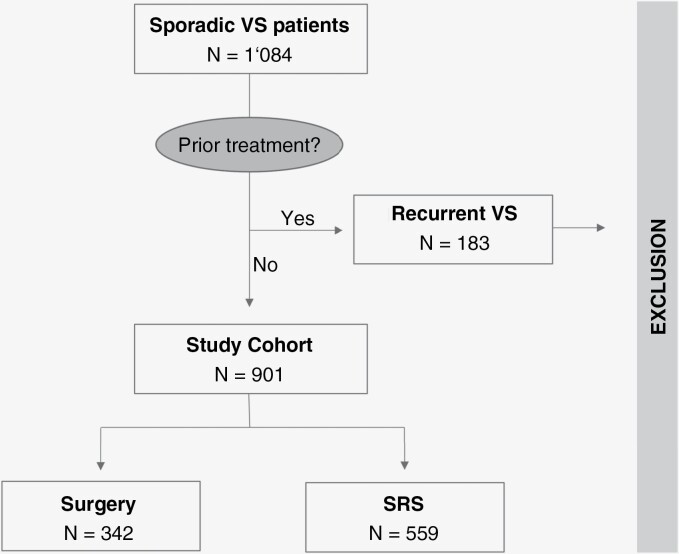
Patient cohort flowchart

Overall, *N* = 1084 patients with VS were treated in both specialized centers within a 6-year period (2005–2011). One-hundred-eighty-three (17%) patients had received previous treatment or suffered from neurofibromatosis type II and were therefore excluded from the study. From the remaining *N* = 901 cases, *N* = 559/901 (62%) tumors were treated with *SRS*, while *N* = 342/901 (38%) tumors were treated with *SURGERY*. A patient flowchart of the cohort is shown in Figure 1.

Overall, the mean age was 54.47(±13.70) years with a significantly older patient cohort in *SRS* at 59.00 (±12.56) years compared to *SURGERY* at 47.45 (±12.48) years (*P* < .001). The *SRS* treatment arm consisted of significantly smaller tumors (Koos I and II) than its surgical counterpart (*P* <.001) (Table 1). Koos III tumors contributed to equal parts to both study cohorts (*SRS*: 36%, *SURGERY:* 38%) (*P* =.499). Of all treated VS tumors, 6% presented with MR-graphic cystic morphology. The proportion was significantly higher in *SURGERY* (9%) compared to *SRS* (4%) (*P* =.002). Demographics are shown in Table 1. Tumor isodose volume was in mean 1.64 (±2.11) cm^3^ with a mean Paddick Index of 0.81 (±0.11) in the *SRS*-treated cohort.

**Table 1.** Patient Demographics, Tumor Characteristics, Clinical Parameters and Treatment Side Effects/ Perioperative Complication Rate and Their Severity. ^*^P-Values Indicate Significant Differences. Values are Presented as the Number of Patients (%) and Mean ± SD, or Median and Interquartile Range Unless Indicated Otherwise. Significant *P*-Values (<.05) are Highlighted in Bold

	ALL (N = 901)	SRS (N = 559)	SURGERY (N = 342)	P-Value
Age	54.47(±13.70)	59.00(±12.56)	47.45(±12.48)	**<.001** ^*^
Female	502 (56)	319 (57)	183 (54)	.297
**Tumor size**				
Koos I	114 (12)	86 (15)	28 (8)	**.001** ^*^
Koos II	295 (33)	213 (38)	82 (24)	**<.001** ^*^
Koos III	330 (37)	200 (36)	130 (38)	.499
Koos IV	162 (18)	60 (11)	102 (30)	**<.001** ^*^
Cystic morphology	55 (6)	24 (4)	31 (9)	**.002** ^*^
**Preoperative Clinical Status**				
Functional hearing (G&R 1–2)	466 (52)	247 (44)	219 (64)	**<.001** ^*^
Good facial function (HB1-2)	886 (98)	546 (98)	340 (99)	**.047** ^*^
Facial spasm	0 (0)	0 (0)	0 (0)	1
Tinnitus	661 (73)	417 (75)	244 (71)	.284
Trigeminus	94 (10)	46 (8)	48 (14)	**.006** ^*^
Vertigo	549 (61)	343 (61)	206 (60)	.737
**Postoperative Clinical Status**				
Functional hearing (G&R 1–2)	216 (24)	134 (24)	82 (24)	1
Good facial function (HB1-2) 1 y	852 (95)	543 (97)	309 (90)	**<.001** ^*^
Facial spasm	29 (3)	29 (5)	0 (0)	**<.001** ^*^
Tinnitus	264 (29)	200 (36)	64 (19)	**<.001** ^*^
Trigeminus	54 (6)	41 (7)	13 (4)	**.030** ^*^
Vertigo	359 (40)	268 (48)	91 (27)	**<.001** ^*^
Treatment complications/side effects	108 (12)	62 (11)	46 (13)	.290
**CDC**				
2	21 (2)	11 (2)	10 (3)	.356
3a	29 (3)	0 (0)	29 (8)	**<.001** ^*^
3b	12 (1)	2 (1)	10 (3)	**.001** ^*^
>4	0 (0)	0 (0)	0 (0)	1

### Clinical Status and Outcome

Pre- and postoperative clinical status is reported in Table 1. The rate of patients with functional hearing at the time of treatment was significantly higher in *SURGERY* (*P* < .001) compared to *SRS*. Vertigo and tinnitus were comparably distinct in both subgroups.

At the last time of the last FU, from all patients, who had good hearing function preinterventionally (*N* = 466), only *N* = 216/466 (46%) had preserved hearing function. Posttreatment hearing preservation was significantly lower in the *SURGERY* group with *N* = 82/219 (37%) compared to *N* = 134/247 (54%) in the *SRS group* (*P* < .001).

Direct significant postoperative facial nerve deterioration (HB > 2) was observed in *N* = 97/342 (28%) in *SURGERY*. However, after 1 year the majority of these patients had improved in facial function, yielding a long-term favorable facial function outcome (H&B 1–2) of *N* = 309/342 (90%). In *SRS*, only *N* = 3 (0.5%) patients suffered from relevant postinterventional facial paresis HB > 2, but *N* = 29 patients suffered from facial spasm, yielding a favorable facial nerve outcome of 95% *N* = 527/559 (95%) if we considered facial spasm as relevant postinterventional facial deterioration. Thus, *SRS* was superior to *SURGERY* in facial function preservation (*P* = .027) in general.

Shunt dependency was indifferent in both treatment groups (*SRS*: *N* = 13/559 (2%) and *SURGERY*: *N* = 6/342 (2%); *P* = .562). *N* = 8 patients suffered from postoperative new trigeminal hypesthesia (*P* < .001) in *SURGERY*. From all patients suffering from trigeminal symptoms, N=72 patients reported improvement or resolution of symptoms after treatment, This rate was significantly higher in the *SURGERY* group with N=46/48 (95%), compared to *N* = 26/46 (56%) in *SRS* (*P* < .001).

The incidence of peri-interventional complications/adverse effects is listed in Table 1 including its CDC Classification. The most common *SRS*-related side effects were symptomatic brain edema or hydrocephalus, while *SURGERY*-related complications were CSF fistula (*N* = 30), hemorrhage (*N* = 5), hydrocephalus (*N* = 5), sinus thrombosis (*N* = 3), symptomatic pneumocephalus (*N* = 2), hygroma (*N* = 2), and infection (*N* = 1). The perioperative complication rate was independent of tumor size (*P* = .623).

When tinnitus was present, patients significantly benefited from surgical resection (*P* < .001); the same was shown in vertigo (*P* < .001). This study reported a higher incidence of worsening tinnitus when treated with *SRS* (*P* < .001) (Table 2).

**Table 2.** Postoperative Secondary Clinical Parameters. ^*^P-Values Indicate Significant Differences. Values are Presented as the Number of Patients (%). Significant *P*-Values (<.05) are Highlighted in Bold

	TINNITUS		VERTIGO	
	*Worsening (N = 83)*	*Improvement (N = 268)*	*Worsening (N = 100)*	*Improvement (N = 276)*
*SRS* *(N = 559)*	69 (12)	77 (14)	69 (12)	130 (23)
*Microsurgery (N = 342)*	14 (4)	191 (56)	31 (31)	146 (53)
*P-value*	**<.001** ^*^	**<.001** ^*^	.128	**<.001** ^*^

### Tumor Control

**Figure 2:**
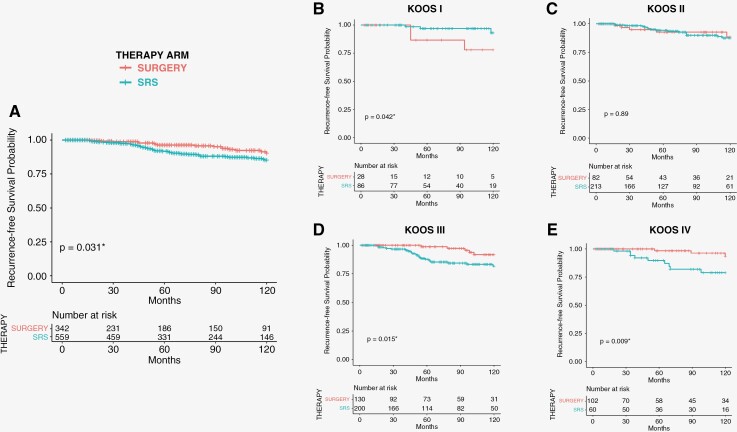
Overall 10-years Kaplan–Meier-analysis shows significantly higher probability of recurrence-free-survival (RFS) in patients treated microsurgically (*P* = .031^*^) in **A**. 10 Years-recurrence-free-survival (RFS) according to **B.** Koos I, **C.** Koos II, **D.** Koos III and **E.** Koos IV. There is a highly significant higher probability for RFS with microsurgical treatment in Koos III and Koos IV VS patients (*P* = .015 and *P* = .009 respectively).

Overall mean postoperative FU was 78.38 (±52.48) months with 82.21 (±52.00) months after *SRS* and 72.12 (±52.72) months after *SURGERY*. The incidence of pseudoprogression was *N* = 177/559 (32%) in the *SRS* ­cohort. Incidence of recurrence (RTC) was reported to be *N* = 84/901 (9%) in the overall study cohort, the incidence of RTC was significantly lower in *SURGERY* (*N* = 25/342; 7%) compared to *SRS* (*N* = 59/559; 11%) (*P* = .032). *N* = 37/59 (62%) of *SRS* recurrences required tumor-specific clinical intervention (Clinical Tumor Control = CTC) (second *SRS*, or *SURGERY* within 1 year). This rate was significantly lower at *N* = 4/25 (16%) in the *SURGERY* cohort (*P* < .001). The mean time to recurrence in VS patients treated with *SURGERY* was 91.07(±40.79) months with 86.84(±44.77) months, when GTR was achieved, and 119.43(±25.98) months in STR. The mean time to recurrence in *SRS* was 64.07 (±38.97) months. Kaplan–Meier-analysis and risk tables are shown in Figure 2.

**Figure 3.**
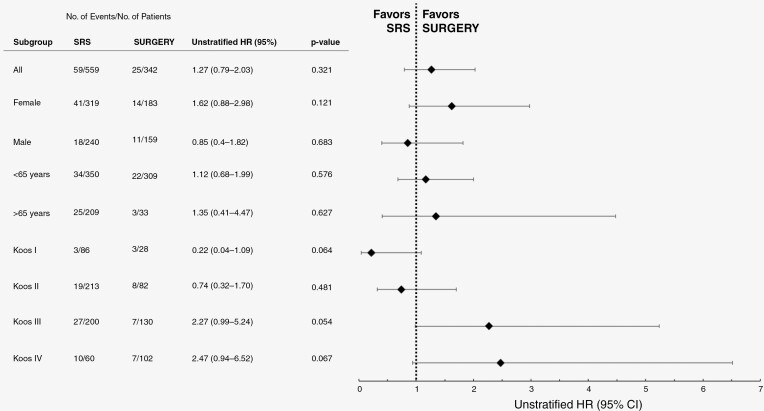
Unstratified hazard ratios for incidence of recurrence in patient subgroups.

In a subgroup analysis of tumor size according to Koos-Classification, RFS was not significantly different in Koos I and Koos II comparing both treatment arms. However, there was a significant benefit of *SURGERY* in regard to RFS for larger tumors (Koos III and IV) (Figure 2). Unstratified hazard ratios for the incidence of radiographic recurrence (radiographic tumor control = RTC) in patient subgroups are shown in Figure 3.

**Figure 4.**
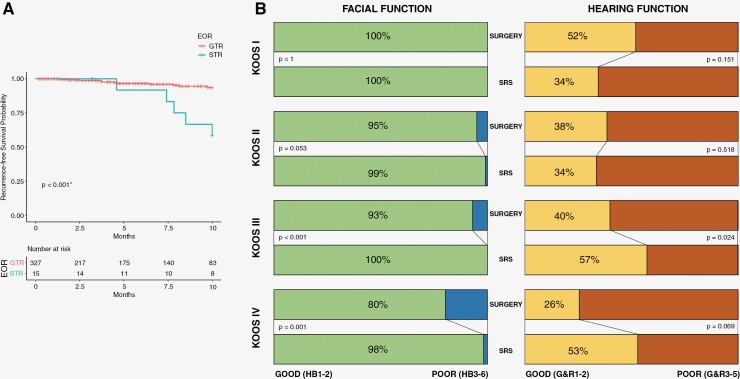
**A.** Shows 10 years-recurrence-free-survival (RFS) according to the extent of resection, **B.** Shows postinterventional functional outcome depending on tumor size (Koos I–IV) classified in good (H&B1–2 and G&R1–2) and poor outcome (H&B3–6 and G&R3–5).

Functional outcomes of hearing and facial function according to tumor size are shown in Figure 4. RFS in the *SURGERY*-group was significantly associated with EOR. STR yielded in earlier tumor recurrences (*P* = .001). However, achievable EOR was significantly impacted by preoperative tumor size (*P* = .013) with *N* = 28/28 (100%) GTR in Koos I, *N* = 81/82 (99%) in Koos II, *N* = 124/130 (95%) in Koos III, and *N* = 94/102 (92%) in Koos IV (Figure 4).

## Discussion

The present study aimed to compare the nuances in the treatment of VS with *SURGERY* and *SRS* in 2 specialized neurosurgical centers. To our knowledge, this is the largest controlled study comparing *SRS* and *SURGERY* as monotherapy in solitary and primary VS. The patient cohort treated with *SRS* was significantly older and consisted of smaller tumors, compared to *SURGERY*. This study reported an incidence of recurrence in *SURGERY* of 7%, and in *SRS* of 11% with a mean FU of 7 years. When comparing both treatment arms, RFS—therefore tumor control—was similar for smaller tumors (Koos I and II). In general, treatment-related functional deterioration (e.g. hearing and facial deterioration) was more common in patients treated with *SURGERY*. What is more, even though the events of treatment-related complications were not significantly different in both groups, *SURGERY*-related complications were classified to be more severe according to CDC (compared to *SRS*). Incidence of recurrence was associated with subtotal resection when treated with *SURGERY* alone. Secondary symptoms like tinnitus, vertigo, imbalance, and trigeminal symptoms were significantly improved by *SURGERY*, but not by *SRS*.

### Primary VS Functional Outcome: Hearing and Facial Function

In either treatment arm, disease-specific mortality was 0% and VS remained a benign tumor with no disease-specific limited life expectancy. Furthermore, clinical outcomes remained satisfactory in both treatment arms.^5^ The rate of patients with functional hearing at the time of treatment (preinterventional) was significantly higher in *SURGERY* (*P* < .001) compared to *SRS*, which may be a result of a significantly younger cohort of the microsurgically treated patients. Long-term postinterventional hearing loss (if the pretreatment hearing function was G&R1–2) was 54% overall, with the significantly highest rate of hearing loss in *SURGERY* (63%), compared to *SRS* (46%). The hearing outcome was comparable in both treatment arms for smaller tumors (Koos I and II). For larger tumors (Koos III and IV), *SRS* was superior to *SURGERY*. Past long-term studies have reported hearing preservation rates of 63–68% at the last follow-up in studies with similar follow-up lengths in *SRS*.^18,19^ However, it remains undocumented, whether even longer follow-up might uncover more delayed cases of hearing loss after *SRS* or even *SURGERY*. Future studies should aim to investigate this aspect of long-term functional outcomes.

*SRS* was superior to *SURGERY* in facial function preservation, especially in large VS, which is concordant to a prospective comparative study by *Myrsethet al*.^20^ Our data showed a direct postinterventional facial paresis was approx. 30% of Koos III and Koos IV VS tumors right after *SURGERY*. However, notably, as reported in the past by Samii et al. in *1997*,^21^ the majority recovered after 1 year in our cohort—yielding a permanent facial paresis HB > II rate of 9.6%. Compared with other available rates of facial function deterioration in the literature from 20% to 46% by retrosigmoid approach, this number reported in this study represents a rather low number (9.6% in the general cohort and 12.5% in large VS Koos III and IV).^19,20,22–24^ The numbers of postoperative facial function outcomes vary largely in the literature, most likely due to the different VS-specific expertise levels and different caseloads between the centers, that have published their data.^19–21,23–25^ In conclusion, surgical and radiosurgical treatment of VS should be carried out in specialized centers with VS-specific expertise, where facial preservation rates posttreatment are the highest. If we regard facial spasm also as a relevant facial affection, *SRS* still yielded a smaller rate of unfavorable facial outcomes at 5.7%.

Several meta-analyses have reported excellent facial function preservation in *SRS*, measured predominantly in HB. Even though facial spasm has been described to be a possible side-effect of *SRS*, this is the first study to report its incidence in comparison with *SURGERY*: Facial spasm is a radiosurgery-specific therapy-related-side effect, with an incidence of 5% in *SRS*, whereas no patient treated with *SURGERY* suffered from facial spasm. Until now, recent literature has focused on facial motor function as a clinical parameter, neglecting facial spasm as a significant compound of SRS-induced facial neuropathy.^9,26^ It is clearly to be debated, whether quality of life is similarly restricted for patients with facial spasm as a facial motor function HB > 2. The true difference or similarity of impact on the everyday life of facial spasm compared to facial paralysis can only be evaluated in a prospective study determining the quality of life in a comparative setting.

### Secondary VS Functional Outcome: Trigeminal, Tinnitus, and Balance

Pre- and posttreatment tinnitus and vestibular function (vertigo, imbalance) are recognized to be fundamental factors that can influence one’s decision in VS management, but are seldom compared in both *SRS* and *SURGERY* treatment arms.^5,9^ When tinnitus was present, patients significantly benefited from surgical resection, which has also been shown by *Wang et al*. in a small prospective study evaluating Tinnitus Handicap Inventory in *N* = 41 with the best prognosis in low-frequency tinnitus.^27^ Our study also reported a high incidence of 12% in worsening tinnitus, when treated with *SRS*. Compared to more recent studies, this data showed an improvement of tinnitus of more than 50% in patients treated with *SURGERY*, this incidence is higher than described by *Trakolis et al*. in 2021^28^ in a similar setting—however, our study uses a more detailed method (grading from grade 1-3 according to symptom severity) to more sensitively describe postinterventional tinnitus dynamic in both *SRS* and *SURGERY* group.

While many studies have focused on hearing, facial function, or quality of life assessment, our data demonstrate that if secondary symptoms (tinnitus, vertigo, etc.) are the main problematic points in the patient’s disease status, *SURGERY* can be evaluated even in smaller tumors. Therefore, physicians have to closely examine, whether these symptoms are present, assess the gravity of these symptoms and discuss the possibility of improvement in case of *SURGERY* during patient consultation.

### Tumor Control

An increased incidence of recurrence was associated with treatment arm *SRS* and in the case of *SURGERY* with lower rates of EOR. The incidence of recurrence, RFS, and mean-time-to-recurrence was significantly worse in *SRS*, showing that *SRS* is inferior to *SURGERY* considering tumor control generally—but especially in Koos III and IV tumors. In small tumors, RFS did not reach statistical significance, therefore from a purely statistical point of view, both treatments (*SRS* and *SURGERY*) are comparable considering tumor control as an outcome parameter. Within the *SURGERY* group, time to recurrence was significantly associated with greater EOR (i.e. GTR), suggesting that residual TV is associated with tumor recurrences. If VS is treated with *SURGERY* alone, safe GTR should be the intended treatment to assure tumor control. Our results are concordant with several series, where a lower extent of resection grades has been shown to be associated with a higher risk for tumor recurrence.^29–31^

Tumor control following *SRS* in large VS is significantly worse compared to smaller VS and microsurgically treated VS of comparable size, which reflects the disease progression rates published in smaller cohorts in the past.^18,32,33^ Accordingly, *Hasegawa et al.* reported in 2005 that worse tumor control was achieved in large VS treated with GKR with a mean-time-to-recurrence below 3 years.^18^ However, the same group later described that tumor regression after 3 years (over 5 years) postinterventionally can be observed.^26^ In conclusion, our data suggest that postinterventional FU investigating tumor control in VS should exceed 5 years even in the *SRS* cohort, as mean-time-to-recurrence was 5.3 years in *SRS* and even longer in *SURGERY*.^18^ Patients treated with *SURGERY* were significantly younger and had larger tumors, which is a phenomenon often described by current literature.^5^ What is more, tumor control was significantly better in young patients and large tumors in subgroup analyses, suggesting that in patients < 65 years of age with large VS, *SURGERY in a center with VS-specific expertise* should be the treatment of choice.

This study did not include patients treated with combination therapy (decompressive surgery followed by adjuvant radiotherapy). However, our dataset shows that *SRS* may not achieve a similar tumor control outcome as *SURGERY* in VS larger than Koos II. In the context of the ongoing discussion about combination therapy (STR or decompressive surgery plus adjuvant *SRS*) in VS, our data add value to the tumor state/size that should be intended to be achieved during surgical decompression.^6^ If we assume that the growth pattern of VS is indifferent after surgical STR, the residual TV should not exceed the tumor category of Koos II, as our data show that tumor control drastically decreases, when VS reaches Koos III (mean TV of 1.57 (±0.87) ccm^3^) when treated with *SRS*. In Conclusion, if combination therapy is chosen—as recommended by the most recent VS management guidelines as Good Clinical Practice Point and other smaller series^6–8^, residual TV after surgical resection should not exceed Koos II, otherwise the patient will be at significantly higher risk for recurrence after adjuvant *SRS* compared to *SURGERY* alone.

The incidence of recurrence of 11% is comparable to the current literature with available comparable incidence rates of 12–15% when the incidence/progression definition was equivalent. Evaluating the effect and tumor control efficacy in 2 modalities so different as *SRS* and *SURGERY* poses a challenge. Residual TV can be higher in noninvasive *SRS* treatment compared to STR, and the difference in size reduction is massive compared to GTR. Detecting tumor recurrence by MR imaging—therefore is more sensitive in *SURGERY* compared to *SRS*. Moreover, the phenomenon of pseudoprogression is only *SRS*-related and can complicate direct comparative efforts immensely.^34^ This phenomenon is observed as studies report a wide range of MR-based recurrence incidence.^19,20,26,35,36^

Although not a primary outcome of this study, the incidence of pseudoprogression of ca. 30% is higher compared to the 23%, which *Hayhurst et al*. reported in the past in a cohort of *N* = 200 between 2005 and 2009 with a median FU of 29 months (2.42 years).^16^ Indeed, the numbers on pseudoprogression in GKR vary from 10% to 71%.^19,26,34,37,38^ This variability most likely results from different FU times (range 29–65 months) and is vividly discussed in the context of the definition of true VS recurrence in *SRS* therapy.^19,37–39^ With a median FU time of 75 months (6.5 years), and retrospective design of this study, no pseudoprogressions are included in the incidence of recurrence as treatment failure, which was defined by sequential growth in serial imaging.

In general, when comparing values in incidence or recurrence, CTC is often used in SRS, which is defined by the necessity for direct clinical intervention, while RTC is more often used in *SURGERY*.^35,40,41^ Therefore, when the comparison is drawn between tumor control rates inter-modally (between *SRS* and *SURGERY*), this has to be taken into account when interpreting any numbers of recurrence. Our study presented an incidence of recurrence in *SRS* of 11%. If the definition is switched to CTC (“necessity for clinical intervention”), 7% (*N* = 37/559) is calculated. Indeed, reported CTC vary from 2% to 12% in GKR studies.^18,35,42,43^ In case of recurrence, the necessity for clinical intervention (within 1 year) was significantly lower in *SURGERY* compared to *SRS*, which is most likely a manifestation of lower residual tumor volume after *SURGERY*.

It is noteworthy that additional studies have shown that the quality of life between treatment groups was not significantly different and *Carlson et al.* described that *SURGERY* may confer an advantage with regard to patient anxiety, presumably relating to the psychological benefit of “cure” from having the tumor removed.^2,44^ Therefore, even though the incidence of recurrence and functional outcome are important statistical statements, the psychological impact of both tumor “cure” and facial paresis must be investigated more thoroughly in the future.

### Limitations of This Study

Although we are providing very detailed clinical information and data in a large study cohort of *N* = 901 identified by prospective registries, the data have been collected in a retrospective manner. Most recently, there was a strong shift in the management of VS away from primary surgery and radiation and toward a “wait-and-scan” approach.^45^ The aspect of the “wait-and-scan”-approach was not included in the study design.

## Conclusions

Our data show that *SRS* can achieve similar long-term tumor control and similar functional results for hearing and facial function to *SURGERY* in smaller VS (Koos I and II)—in combination with less severe postinterventional morbidities. If secondary VS symptoms (e.g l tinnitus, vertigo/imbalance, and trigeminal symptoms) are severe—even in small VS—*SURGERY* can be recommended, as *SURGERY* may more likely improve these symptoms, but *SRS* may not. In Koos III and IV VS, functional results of hearing and facial function are superior in *SRS*, still, the effect of *SRS* on tumor control is inferior compared to *SURGERY*. Therefore, especially in the young population and large VS, *SURGERY* should be favored to *SRS*. If combination therapy is chosen, the residual tumor should not exceed the size of Koos II.

## Data Availability

All data and materials are available and can be provided upon reasonable request.
